# iGEMS: an integrated model for identification of alternative exon usage events

**DOI:** 10.1093/nar/gkw263

**Published:** 2016-04-19

**Authors:** Sanjana Sood, Krzysztof J. Szkop, Asif Nakhuda, Iain J. Gallagher, Carl Murie, Robert J. Brogan, Jaakko Kaprio, Heikki Kainulainen, Philip J. Atherton, Urho M. Kujala, Thomas Gustafsson, Ola Larsson, James A. Timmons

**Affiliations:** 1 Division of Genetics and Molecular Medicine, King's College London, WC2R 2LS, London, UK; 2 Research Department, XRGenomics Ltd, 35 Kingsland Road, London E2 8AA, UK; 3 School of Medicine, University of Nottingham, Derby Royal Hospital, Derbyshire, DE22 3DT, UK; 4 School of Health Sciences, University of Stirling, Stirling, FK9 4LA, Scotland; 5 Department of Oncology-Pathology, SciLifeLab, Karolinska Institutet, 171 76 Stockholm, Sweden; 6 Department of Public Health and the Institute for Molecular Medicine (FIMM), University of Helsinki, FI-00014, Helsinki, Finland; 7 National Institute for Health and Welfare, University of Helsinki, FI-00014, Helsinki, Finland; 8 Department of Biology of Physical Activity, University of Jyväskylä, FI-40014, Jyväskylä, Finland; 9 Department of Health Sciences, University of Jyväskylä, FI-40014, Jyväskylä, Finland; 10 Department of Laboratory Medicine, Division of Clinical Physiology, Karolinska University Hospital, 14186, Huddinge, Sweden

## Abstract

DNA microarrays and RNAseq are complementary methods for studying RNA molecules. Current computational methods to determine alternative exon usage (AEU) using such data require impractical visual inspection and still yield high false-positive rates. Integrated Gene and Exon Model of Splicing (iGEMS) adapts a gene-level residuals model with a gene size adjusted false discovery rate and exon-level analysis to circumvent these limitations. iGEMS was applied to two new DNA microarray datasets, including the high coverage Human Transcriptome Arrays 2.0 and performance was validated using RT-qPCR. First, AEU was studied in adipocytes treated with (*n* = 9) or without (*n* = 8) the anti-diabetes drug, rosiglitazone. iGEMS identified 555 genes with AEU, and robust verification by RT-qPCR (∼90%). Second, in a three-way human tissue comparison (muscle, adipose and blood, *n* = 41) iGEMS identified 4421 genes with at least one AEU event, with excellent RT-qPCR verification (95%, *n* = 22). Importantly, iGEMS identified a variety of AEU events, including 3′UTR extension, as well as exon inclusion/exclusion impacting on protein kinase and extracellular matrix domains. In conclusion, iGEMS is a robust method for identification of AEU while the variety of exon usage between human tissues is 5–10 times more prevalent than reported by the Genotype-Tissue Expression consortium using RNA sequencing.

## INTRODUCTION

RNA splicing occurs in all-eukaryotic organisms and requires a sophisticated machinery to correctly define exon boundaries for the removal of introns (1). Splicing can give rise to diverse transcripts with alternative patterns of exon inclusion/intron retention altering protein function or post-transcriptional regulation (2). Alternative splicing (AS) or alternative exon usage (AEU) occurs in most mammalian multi-exon genes (3,4) and alterations in AEU has been linked to a number of diseases, including several cancers (5–8) and neurodegeneration (9). AEU that lead to pathology, or altered physiological regulation can be studied using various global transcriptomics techniques. These include RNA sequencing (RNA-seq) (10) and DNA microarrays such as Affymetrix Exon ST arrays (11) and Human Transcriptome Arrays 2.0 (HTA 2.0). The Sequencing Quality Control Consortium (SEQC) recently concluded that deep RNA-seq in combination with high-resolution DNA microarrays may provide the most efficient approach for studying the transcriptome (12), recognizing the complementary nature of the two technologies. The strength of sequencing technology is that it aspires to capture the entire diversity of the transcriptome.

Microarrays have several potential advantages over sequencing, particularly for quantifying lower abundance transcripts. Hybridization technologies typically rely on greater amounts of cDNA, than RNA-seq. However, the array-based detection of each cDNA (or cRNA) is independent, so avoiding the competitive detection scenario which limits the performance of sequencing. For example, the inability of sequencing low abundance transcripts reflects highly abundant RNAs accounting for a very large proportion of cDNA library (13) so limiting the diversity of the library. This limitation will in turn lead to inefficient assessment of the variation in exon use. Using RNA-seq the Genotype-Tissue Expression (GTEx) consortium recently concluded that each human tissue transcriptome was defined by relatively few unique genes and surprisingly limited AEU across tissues (14).

It is plausible that this conclusion reflects technical limitations rather than genuinely sparse use of AEU. However it may also reflect limitations of the computational methods used to identify splicing. Indeed most algorithms for identification of AEU are infrequently utilized because their performance is suboptimal. For RNA-seq, more frequently used methods include ‘Mats’, (15) or ‘DEXSeq’ which implements a generalized linear model to identify AS (16). Cufflinks (17) is another commonly used approach focused on transcript assembly and differential isoform expression analysis. Unfortunately, none provide clear evidence of yielding a high true-positive detection rate (18) and therefore improved AEU models are needed.

Surprisingly, entirely satisfactory analytical pipelines for accurate identification of AEU using microarray data also remain to be established (18,19). Early pipelines for analysis of data acquired using DNA microarrays include splicing index (SI) (20), Microarray Detection of Alternative Splicing (MiDAS) (21), Pattern-Based Correlation (PAC) (22), Analysis of Splice Variation (ANOSVA) (23) and correlation coefficients (24). More recent methods include ARH (25), Multiple Exon Array Preprocessing (MEAP) (26), Corrected Splicing Indices for Exon Arrays (COSIE) (27) and Finding isoforms using Robust Multichip Analysis (FIRMA) (28). Given the complexity of the transcriptome and the substantial potential for generating abundant false positive results, all of these methods require laborious manual evaluation of the outputs.

The SI method adjusts exon-level expression values by the corresponding gene-level value and then compares individual exons across two sample classes (20). MiDAS (See Affymetrix technical support document, ‘exon_alt_transcript_analysis_whitepaper.pdf) is conceptually similarly to SI in as much as it incorporates an analysis of variance (ANOVA) model across multiple sample groups. However, as the number of exons per gene increases, the reliability of MiDAS decreases as it lacks robust control for multiple testing (25). The most frequently cited method applied to Exon array data is FIRMA, later adapted to FIRMAGene for Affymetrix Gene ST platform (28,29). FIRMA fits a linear model to a composite transcript cluster for each gene, using the residuals to indicate if the model, and hence gene, has changed between two conditions. FIRMAGene (29) arranges the residual values into genomic order such that persistent and consistent change in residuals is used to indicate that a particular gene has AEU (as defined by a maximum score per gene), making FIRMAGene a more powerful model than FIRMA. The FIRMAGene R package does not, however, adjust for multiple testing (a non-trivial issue given the impact of gene size), nor does it identify the precise location of the AEU event(s), making extensive visual inspection obligatory.

Validation of the aforementioned methods has largely relied on contrasting RNA expression between two diverse tissues, an extreme scenario of AEU likely resulting in a focus on easier-to-detect examples of AEU (30). Therefore, this type of validation provides limited basis to conclude that an AEU method would be sensitive enough to identify splicing events within a cell responding to more subtle changes in experimental conditions (31,32). Our aim was therefore to develop and validate a pipeline that returned a high true positive rate for AEU while being sensitive enough to allow identification of AEU under conditions where the cell is responding to routine experimental conditions. Here we introduce iGEMS, which is a development of the FIRMAGene approach, introducing a gene-size adapted p-value adjustment in combination with a false-positive filtered exon level metric that removes the need for visual inspection. We applied iGEMS to two new DNA microarray data-sets; a primary adipocyte model treated with rosiglitazone (ROSI, a PPARG agonist (33)) representing a typical cell biology experiment and a newly generated inter-tissue analysis using the HTA 2.0 gene-chip technology. The technical validation rate was high and iGEMS identified many more AEU events than recent RNA-seq based studies of the human tissue transcriptome.

## MATERIALS AND METHODS

### Cell culture, drug treatment and cell culture DNA microarray data

Primary adipocyte progenitors were isolated from inguinal subcutaneous tissue obtained from SV129 and C57BL/6 mice (Harlan, UK). Tissue was processed as previously described (34–36). Precursor cells were suspended in culture media and cultured in six-well plates containing Dulbecco's modified Eagle's medium with 10% (vol/vol) newborn calf serum (Life Technologies), 2.4 nM insulin, 25 μg/ml sodium ascorbate, 10 mM HEPES, 4 mM glutamine, 50 U/ml penicillin and 50 μg/ml streptomycin, supplemented or not (as indicated) with 1 μM rosiglitazone maleate (Enzo Life Sciences) from the first day of culture leading to a robust metabolic phenotype shift in these cells (35). The cells were cultured at 37°C in an atmosphere of 5% CO_2_ in air with 80% humidity. Before harvesting, cells were examined using phase contrast microscopy (on Leica DMIRB inverted microscope) to confirm health, while the media was changed on day one and every second day, but not on the day the cells were harvested.

Total RNA was isolated from the cultured primary adipocytes on day 7, using TRizol^®^ (Life Technologies) as previously described (37) and quality/quantity was checked using NanoDrop 2000 spectrophotometer. RNA was dissolved in 20 μl RNAse-free water with no additives. Affymetrix exon arrays were processed according to the manufacturer's protocol. Five microgram of fragmented, end-labelled sense strand target cDNA was hybridized to each Mouse Exon 1.0 ST array and a Gene Chip Scanner 30007G was used to scan the arrays (BEA Core facility, Huddinge Hospital, Sweden). From the 20 RNA samples processed, three samples were excluded as outliers by visually evaluating principal component analysis (PCA) and normalized unscaled standard error plots prior to any down-stream analysis. The raw data has been deposited at GEO (GSE57903).

### Human tissue and DNA microarray data

Tissue from monozygotic twin pairs from the FITFATTWIN study (FinnTwin16 Cohort) - a population based, longitudinal study of Finnish twins born between October 1974 and December 1979 was used in this study (38). Selection of the twin pairs for the FITFATTWIN study was performed on the basis of a web-based questionnaire survey to the cohort (39), telephone interview, interview at the laboratory and medical examination at the laboratory (40). The participants had no chronic disease affecting the ability to exercise, no acute disease and no drug or alcohol abuse and thus can be considered healthy (Table 1). All experimental procedures and study protocols were approved by the Ethical Review Board for Human Research of the Central Finland Health Care District (29 September 2011) and the study was conducted in accordance with the Declaration of Helsinki. All participants volunteered and provided written informed consent.

**Table 1.** Twin study clinical characteristics

	Mean ± SD	Min	Max
Age (y)	34 ± 1.45	32	37
Height (cm)	1.78 ± 0.07	1.6	1.9
Weight (kg)	76.51 ± 9.8	51.3	95.9
Body mass index (kg/m^2^)	24.12 ± 2.7	19.8	33.6
Waist circumference (cm)	86.19 ± 7.8	70.5	111.5
Fat percent (%)	21.47 ± 6.8	7.7	35.9
Systolic blood pressure (mmHg)	113.34 ± 9.9	94	140
Diastolic blood pressure (mmHg)	68.8 ± 7.5	50	80
Fasting glucose (mmol/l)	5.47 ± 0.5	4.7	6.6
Fasting insulin (μU)	3.85 ± 3.2	0.2	14.6
HOMA index	0.96 ± 0.87	0.04	4.3

A summary of the clinical characteristics of the male monozygotic twins (*n* = 14) with adipose, muscle and blood samples that were profiled on the human transcriptome 2.0 array.

Various clinical parameters were collected, as previously described (40). Briefly, measurements of physical fitness, body composition (including fat percentage measured by dual-energy x-ray absorptiometry, (DEXA) and blood samples including glucose homeostasis from an oral glucose tolerance test) were collected. Body composition data included BMI (mass (kg) / height (m)^2^) and waist circumference. Whole body DEXA was performed (DEXA Prodigy; GE Lunar Corp., Madison, WI, USA) to estimate lean tissue and body fat mass. VO2max (ml/min) was determined by bicycle ergometer exercise test using the mean of the two highest 30s recordings at the end of the max test. Vmax spiroergometer (Sensormedics, Yorba Linda, CA, USA) was used to measure oxygen uptake and gaseous exchange. Supine blood pressure (mmHg) was recorded in the morning prior to VO2max test, during VO2max test and at 2 min intervals during recovery. Fasting blood samples were taken following an overnight fast and 10 min of supine rest upon arrival. Participants then underwent a 75 g oral glucose tolerance test with blood(s) samples taken at thirty minutes, 1 and 2 h post glucose load. Serum glucose and insulin levels were measured by Konelab 20 XT (Thermo Fisher Scientific, Vantaa, Finland) and IMMULITEÒ 1000 Analyzer (Siemens Medical Solution Diagnostics, Los Angeles, CA, USA) respectively. Metabolic health was defined by the homeostatic model assessment (HOMA) (41).

Blood (PaxGene), muscle and fat were collected for RNA isolation. Procedures for taking the muscle and adipose tissue biopsies were as before (42), with tissue samples taken after an overnight fast between 8 and 10 am under local anesthesia. The muscle biopsy was taken from the mid-part of *m. vastus lateralis* using a Bergström's needle (ø5 mm) and a needle biopsy (12 G needle, ø2 mm) of subcutaneous abdominal adipose tissue was taken at the level of the umbilicus. The samples were cleaned of any visible connective tissue and muscle samples were cleaned of any visible adipose tissue. One part of each tissue biopsy was frozen in liquid nitrogen immediately after withdrawing from the needle and stored at −80ºC until used for analysis. For the twin tissue (muscle, blood and adipose) gene-chip analysis, RNA was isolated using TRizol^®^ (Life Technologies) as previously described (37) and quality/quantity was checked using NanoDrop 2000 spectrophotometer (Labtech International, UK). RNA was dissolved in 20 μl RNAse-free water (Life Technologies) with no additives. Affymetrix HTA 2.0 DNA microarrays were processed according to the manufacturer's protocol. Five microgram of fragmented, end-labelled sense strand target cDNA was hybridized to each DNA microarray and a Gene Chip Scanner 30007G was used to scan the DNA microarrays (Affymetrix Core, MPI A/S, Denmark). From the 70 RNA samples processed, nine samples were excluded as outliers by visually evaluating PCA and normalized unscaled standard error plots prior to any down-stream analysis. The raw data has been deposited at GEO (GSE73142). For the purposes of the present analysis we did not implement use of the twin structure (each pair were discordant for environmental factors) as we focused on paired tissue comparison within individuals.

### Description of the iGEMS multi-step alternative exon usage identification pipeline

**Figure 1.**
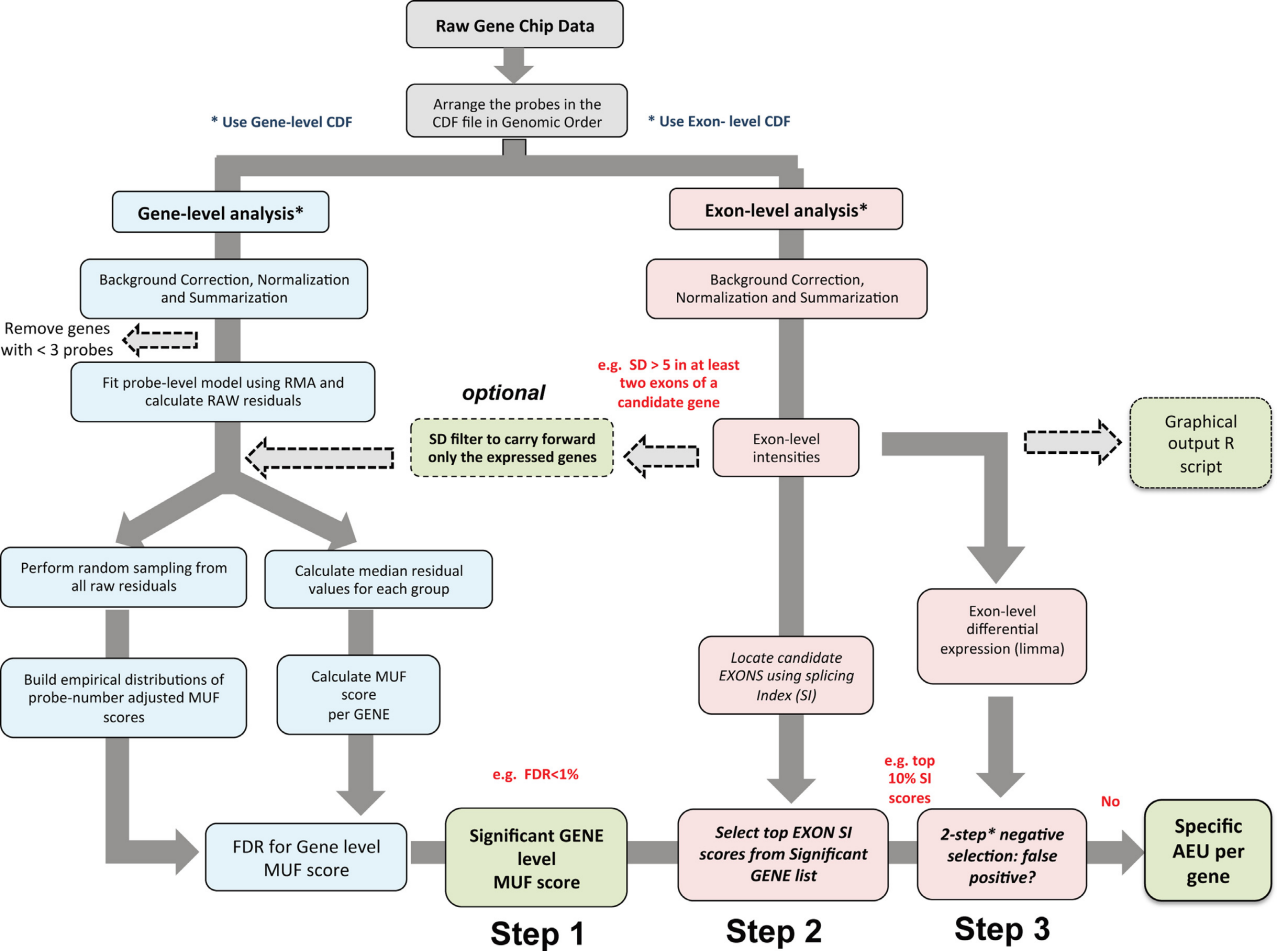
Schematic diagram of iGEMS pipeline for the identification of alternative exon usage (AEU). iGEMS integrates analysis at both at the gene (blue boxes) and at the exon level (red boxes). An optional filter based on standard deviation can be applied to remove genes likely to be non-expressed which is important in scenarios where gene expression is absent in one condition. At the gene level we calculate the FDR for the adjusted Material Unaccounted For (MUF) score. Genes with significant MUF score (FDR < 1%) are analysed further (Step 1). To locate the AEU within candidate genes (i.e. genes with a significant absolute MUF score) we calculate a splicing index (SI) per exon. Genes with at least one exon with an SI value in the top or bottom decile of the SI distribution of the Step 1 filtered data are selected (Step 2). In the third iGEMS step we apply a ‘negative selection’ filter, which identifies exons as false positive AEU events if expression of the exon, in both the control and treatment groups, is lower than the median gene expression for respective conditions, and where the associated Limma based FDR indicates that the respective exons are *not* differentially expressed (FDR > 1%) (Step 3). This yields a modest filtering of the genes selected at steps 1 and 2 (FDR adjusted MUF scores and SI ranked list) and results in a list of genes that undergo an AEU.

Aroma.affymetrix (43) and Bioconductor (44) packages were used to develop an integrated pipeline for ‘automated’ identification of AEU (Figure 1). The strategy was to produce gene-level and exon-level based ranking of genes, while adjusting for multiple testing. The aim was to yield a high true positive rate for selected AEU events while limiting ad-hoc or complex heuristic-sensitive filtering processes. The procedure for implementing FIRMAGene was adapted (29) for use with Exon Array ST arrays and an updated CDF (chip definition file), representing Ensembl gene identifiers, was sourced from BrainArray (45) (Figure 1). Probes were arranged in genomic order and probe-set expression values were calculated using RMA (46). Probe-sets with less than three probes were removed from the analysis as these are not considered to provide a very reliable measure of transcript expression. The equations detailing the Robinson and Speed FIRMAGene model (29) are shown in Supporting File: Supporting Information 1.1. In contrast to the original implementation, we used median residual values, rather than standardized residuals, to estimate deviation from the FIRMAGene model across biological replicates as median is more robust to the influence of outliers. The maximum absolute partial sum of residues is computed per gene, a parameter defined by Robinson *et al*. to as the ‘Material Unaccounted For’ (MUF) score (28).

The difference between the median residual values between two conditions (e.g. control versus ROSI treated primary adipocytes) reflects, in our study, the impact of drug treatment on AEU, and a high absolute MUF score indicates stronger gene-level evidence for AEU. However, as the number of probes per gene increases, there are more partial sums to consider and more extreme MUF values will occur by chance. We therefore implemented a false discovery rate (FDR) approach (47). Empirical distributions of MUF values, for different numbers of probes, were achieved by randomly sampling from all available residuals (so adjusting for ‘gene size’ (variations in exon number)). FDR values for experimentally observed MUF scores can then be derived by calculating the probability of obtaining an identical or more extreme MUF score from the empirical distribution. This was implemented using the ‘stats’ package in R, and an FDR threshold is then chosen above a given quantile (e.g. 1% FDR). This represents ‘**Step 1**’ in the AEU identification (Figure 1).

However, identification of a *gene* with a significant MUF score does not identify the AEU event. For this we implemented the SI method (equations for this method in Supporting File: Supporting Information 1.1, adapted to include a false positive filter (See below)), to identify the likely location of an AEU event. Incorporation of this measure allows ranking of exons within genes that have a significant MUF score. In the cell culture experiment, genes with an SI value in the top or bottom decile of the SI distribution represents the second step of AEU identification (denoted as ‘**Step 2**’ in Figure 1). As this was a method development project, visual validation was used to evaluate performance and thus expression values and residual plots were generated for genes that passed the step 1 and 2 criteria (Figure 1). Biomart (48) was used to link expression data to the latest version of identifiers in Ensembl while RMA normalized data and residuals were plotted using the ‘GenomeGraphs’ package.

During the visualization validation process we noted genes with robust MUF and SI scores originating from exons where the *preceding* exons in both the control and drug-treated groups had expression values that approximated background. A simple ‘detected as expressed’ filter, which attempts to estimate a threshold for background expression (49), removed a substantial number of true AEU events and thus was not useful. Instead, an exon-level filter was developed where lack of differential *exon* expression (50) between the two groups on the AEU location was used to detect the aforementioned class of false positives. More specifically, for a false positive SI event, we identified exon(s) where expression in both the control and treatment groups was *lower* than the median gene expression and where the exon preceding the significant SI event was *not* differentially expressed between conditions (e.g. >1% FDR in the present dataset, using limma). This reduced the number of genes identified from the FDR adjusted MUF score and SI ranked list (i.e. AEU identification steps **1** and **2**) by ∼25%. This step is denoted as ‘**Step 3**’ in Figure 1. Convincingly, visual inspection of a large sample of the genes removed by the third step indicated removal of >90% genuine false positives (also supported by RT-qPCR (see results)). Finally an optional low-level filter to eliminate non-expressed genes can be implemented using a small absolute value for gene expression standard deviation (SD), with the value chosen reflecting the individual gene-chip and experimental characteristics (See tissue analysis section below).

### Experimental validation of murine AEU events by RT-qPCR

For RT-qPCR 500 ng of total RNA was reversed-transcribed with the High-Capacity cDNA Reverse Transcription kit (Life Technologies) according to manufacturer's protocol. cDNA was diluted 1:5 and 1 μl was added per well to 384-optical well plates (Life Technologies). Exon specific primers (Sigma-Aldrich) were designed for genes selected from the first 100 ranked genes, with Primer-BLAST. Primers were mixed with SYBR Select Master Mix (Life Technologies) and aliquots of 11 μl of mastermix were added to each well. Samples were run in triplicate. Thermal cycling conditions were 2 min at 50°C followed by 2 min at 95°C, and 40 cycles of 15 s at 95°C and 60 s at 60°C on a ViiA™ 7 Real-Time PCR System (Life Technologies). To control for RNA input, 18S levels were measured using an Eukaryotic 18S rRNA Endogenous control kit (Life Technologies) according manufacturer's protocol with 1:200 diluted cDNA. Target mRNA expression was quantified using the ΔC_T_ method (51). For each gene an AEU exon and an exon deemed constitutively expressed was measured. The data is presented as percentage change (%) from control. To determine the statistical significance a Mann–Whitney U test was preformed on the ΔC_t_ values followed by Bonferroni correction. Primers used for the murine Exon array RT-qPCR validation can be found in Supporting File 2: Supplementary Table S1. Transcript variants were visualized using GPViz (52). Methods for HTA 2.0 RT-qPCR validation can be found in Supporting File 1: Supporting Information 1.2.

### Functional characterization of AEU

There are limitations to the usefulness of pathway or gene ontology (GO) analysis for identifying enrichments of biological functions among genes in a list of regulated genes (53). The ‘regulated gene list’ (in this case genes with AEU events) must be contrasted with a list of the *detectable* genes in the experiment (and not all the genes in the genome or on the gene-chip) (32,54). We analysed the list of genes containing AEU events to determine if that list was enriched in GO categories that either supported the known biological differences between any two tissues, or included molecular functions related to functional protein features (as exon removal/inclusion is proposed to alter protein structure/function). We utilized GOstats package in R with current GO categories (GO.db) to examine GO hierarchy and identify the point of greatest enrichment using R (55). We characterized the impact of the change in protein sequence, brought about by AEU. The exon in each significant gene, with the most positive and most negative SI, was mapped to amino acid ranges using the expected products of coding transcripts. The exon based amino acid ranges were then overlapped with Pfam domain positions (i.e. envelope_start, envelope_end) using data from Pfam release 29 (56). The Pfam domain frequency was plotted for each inter-tissue comparison.

## RESULTS

### Implementation of iGEMS pipeline using the Affymetrix EXON ST platform

**Figure 2.**
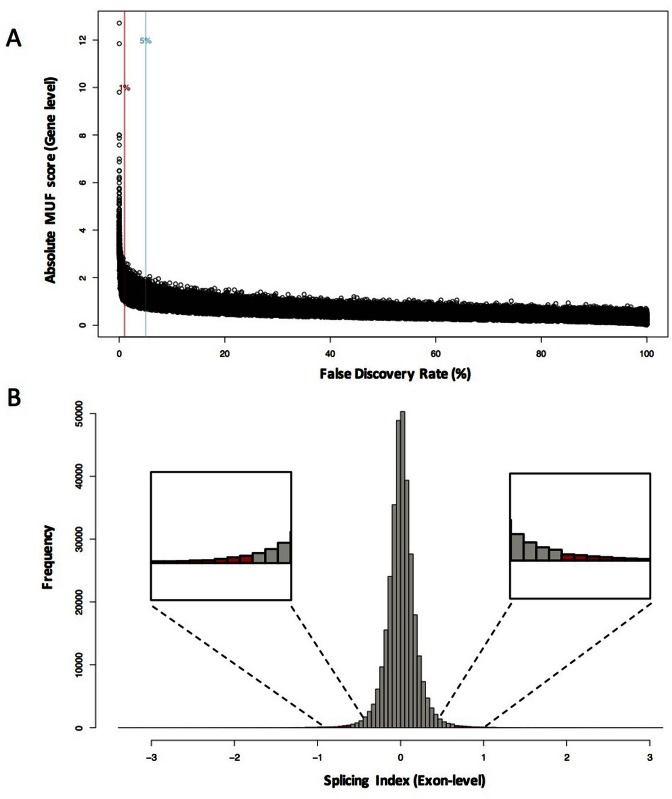
Distribution of absolute MUF scores (step 1) and SI (step 2) in adipocytes treated with rosiglitazone and profiled using Affymetrix Mouse Exon 1.0 DNA microarrays. (**A**) The distribution of MUF scores (absolute) and the corresponding FDR values. The red and blue line represents the 1% and 5% FDR cut-off values respectively. Using the 1% cut-off we identified 1464 genes to have significant MUF score. (**B**) Shown is the distribution of SI calculated at the exon-level for all exons. Using the exons from the 1464 candidate genes (∼22 652 exons) we apply the upper and lower decile for the SI cut-off as −0.635 and 0.646 respectively. This resulted in 2266 exons (red bars) passing step 2 (Figure 1), which corresponded to 729 genes as candidates for AEU.

For development of iGEMS we generated a dataset contrasting cells with or without ROSI treatment (see methods), i.e. where more subtle changes in AEU are expected as compared to comparisons between diverse tissues. The outline of the iGEMS pipeline is shown in Figure 1 and summary statistics of the processing of transcriptomics data in Table 2. The R script for each chip-type specific iGEMS pipeline is provided at https://github.com/iaingallagher/iGEMS_scripts. The MUF scores and the associated FDRs for each gene were calculated, contrasting control with ROSI treatment. As expected the relationship of FDR to MUF scores did not follow a simple linear trend because, depending on the number of probes for a gene, a specific MUF score will be associated with different FDRs (Figure 2A). We selected those genes with a MUF score associated FDR<1% (Figure 2A). This resulted in a list of 1464 genes with evidence for AEU in response to ROSI. To identify which exons are responsible for these gene-level scores, we calculated the SI for each of the 22 652 exons present in our candidate 1464 gene list. Approximately 50% of the genes with significant MUF scores also contained examples of exons with more extreme SI scores (*n* = 729, <1% FDR, Figure 2B).

**Table 2.** Summary statistics of the alternative exon usage (AEU) pipeline on Mouse Exon 1.0 ST array

Group	Filtering steps of iGEMS	Genes or exons
**A**	Number of exons annotated on array	325 446
**B**	Number of genes annotated on array	26 500
**C**	Number of genes with <5% FDR	4688
**D**	Number of exons represented from **C**	69 253
**E**	Number of genes with <1% FDR	1464
**F**	Number of exons represented from **E**	22 652
**G**	Number of genes from **E** within 10% SI distribution	729
**H**	Number of exons from **F** within 10% SI distribution	2266
**I**	Number of genes after removing false positives from **G**	555
**J**	Number of exons after removing false positives from **H**	1677

Flow of the transcriptomic data through the stages of the analysis workflow, starting with all the probe-set features on the Mouse Exon 1.0 ST array measured using gene and exon BrainArray CDF files. Using the AEU pipeline on the murine primary adipocyte gene- and exon-level expression data, a final list of 555 genes with evidence for AEU was identified.

**Figure 3.**
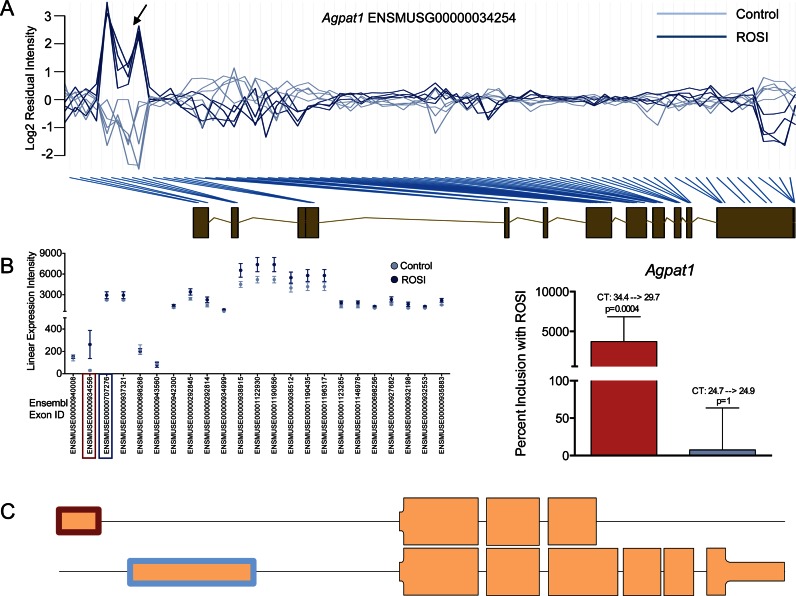
Identification of *Agpat1* undergoing an AEU event in response to rosiglitazone (ROSI). (**A**) The MUF score is derived from the residual plot of *Agpat1* (ENSMUSG00000034254). Residual values were plotted in genomic order with the composite gene structure juxtaposed below. Our analysis was performed on eight control (light blue) and nine ROSI treated cell cultures (dark blue). Blue lines connect the residual value to their respective genomic regions. Towards the 5′ end of *Agpat1* there was a high deviation from the model indicating an AEU event (black arrow). (**B**) Expression plot of the mean expression intensity (±standard deviation (SD)) of each Ensembl exon ID assigned to *Agpat1*; light blue represents the control and dark blue the ROSI treated values. Ensembl Exon ID ‘ENSMUSE00000934556′ is the AEU exon (red box) and ENSMUSE00000707276 (blue box) is the constitutively expressed exon. Primers were designed to independently measure these two exons. RT-qPCR validation was carried out in independent RNA (mean ± SD; control (*n* = 8) and rosiglitazone (ROSI) (*n* = 9)) and is shown as percent (%) change from the control group with ROSI with the colour consistent with the exon ID plot on the left. CT values and adjusted *P*-values are shown for the control and ROSI group. (**C**) Schematic of the two *Agpat1* variants: a full *Agpat1* variant (ENSMUST00000037489, lower panel) which does not contain the AEU exon we identified, and the truncated *Agpat1* (ENSMUST00000173242, higher panel) which contains the AEU exon. The highlighted exons (red and blue) represent the AEU exon and the constitutively expressed exon, respectively as measured by the array and the RT-qPCR primers. Exons part of the translated RNA is represented as a thick box than untranslated.

Figure 3A shows the residual plots for *Agpat1* (ENSMUSG00000034254) with a strong spike in residuals towards the 5′ end of the gene (black arrow). The relatively simple task of identifying AEU in *Agpat1* can be contrasted with *Ptprd* (Supplementary Figure S1) where visual inspection is not practical. We selected *Agpat1* and six additional genes for RT-qPCR validation. An extreme SI was used to select the exon(s) undergoing AEU and this was contrasted with an exon that had relatively stable expression between groups (Figure 3B). For *Agpat1* RT-qPCR primers were designed (Supporting File 2: Supplementary Table S1) to target exon ENSMUSE00000934556, the AEU exon, while a second set of primers targeted to Exon ENSMUSE00000707276 (blue box), the constitutively expressed exon (Figure 3B and C). Indeed RT-qPCR confirmed that ENSMUSE00000934556 was subject to AEU by ROSI (Figure 3B, increased ∼5 CT values, *P* = 0.0004) while the constitutively expressed exon remain unchanged. It should be noted that the accompanying plot of exon expression values, derived from microarray data are not corrected for probe-characteristics (e.g. nucleotide composition etc.) and thus absolute expression values are not directly comparable across exons.

**Figure 4.**
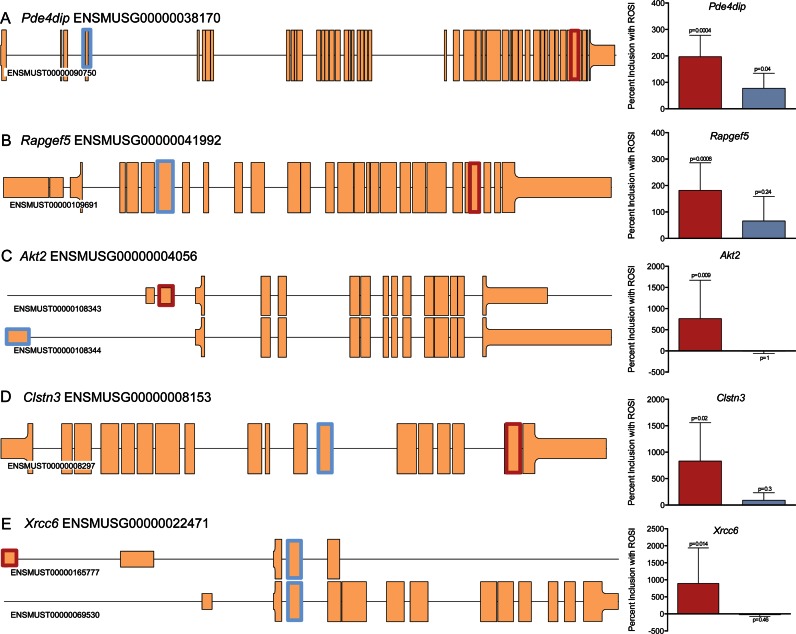
Validation of five AEU events by visual inspection and RT-qPCR. For each gene, a schematic of the transcript(s) is shown with red and blue boxes highlighting the AEU exon and constitutively expressed exon, respectively (targeted by RT-qPCR primers). Exons part of the translated RNA is represented as a thick box than untranslated. RT-qPCR data is shown as percent (%) change from the control group (*n* = 8) with rosiglitazone (ROSI) (*n* = 8) (mean ± standard deviation) with the colour consistent with the Exon ID plot above. The expression of AEU exon changed relatively more than the constitutively expressed exons, in response to ROSI treatment. (**A**) *Pde4dip* (ENSMUSG00000038170), (**B**) *Rapgef5* (ENSMUSG00000041992), (**C**) *Akt2* (ENSMUSG00000004056), (**D**) *Clstn3* (ENSMUSG00000008153) and (**E**) *Xrcc6* (ENSMUSG00000022471).

The same approach was applied to six additional genes (Figure 4 and Supplementary Figure S2; exon expression values are shown in Supplementary Figure S2) identified by the first two steps in our pipeline and all but one validated. Thus, we validated *Pde4dip* (ENSMUSG00000038170, Figure 4A), *Rapgef5* (ENSMUSG00000041992, Figure 4B) *Akt2* (ENSMUSG00000004056, Figure 4C), *Clstn3* (ENSMUSG00000008153, Figure 4D) and *Xrcc6* (ENSMUSG00000022471, Figure 4E). *Rxrg* (ENSMUSG00000015843, Supplementary Figure S3) failed to validate and the residual plot for *Rxrg* demonstrated a high MUF score (Supplementary Figure S3A) coupled with changes in global expression, but not at the 5′ end as there was an apparent lack of expression of the 5′ exons (red box, Supplementary Figure S3B). RT-qPCR confirmed that the proposed AEU event was a false positive, caused by the 5′ part of the signal approximating background expression i.e. not expressed (e.g. RT-qPCR CT values >37). In contrast, ENSMUSE00000687625 (blue box, Supplementary Figure S3B) was robustly expressed and increased in response to ROSI treatment (*P* = 0.0002).

Thus sequential changes in exon expression preceded by a ‘signal’ from non-expressed can lead to false positive AEU events. As a general solution for this problem we applied a negative selection criterion as described above (Step 3, an exon level false positive filter (Figure 1). We confirmed that this simple filter accurately removed ∼90% of such false positives, including *Rxrg*, using extensive visual inspection, yielding a final list of 555 AEU genes from the original list of 729 (Table 2). Notably, none of the laboratory validated AEU events were removed by this **Step 3**. In summary, by combining gene level and exon level analysis with a simple filter for non-expressed probe-sets, iGEMS efficiently identified AEU with a low occurrence of false positives, our primary aim. iGEMS is not specifically optimised for identification of all true-positives, as such an aim will be more heavily influenced by sample size and size effect (the magnitude of AEU).

### Application of the iGEMS pipeline to Affymetrix human tissue array 2.0 data

Analysis of muscle, adipose and blood samples using the HTA 2.0 array allowed for a direct comparison with GTEx consortium transcript diversity analysis (14) of these same tissues. Our samples originated from monozygotic twins (40) of a very similar age (∼33 years) and in good health (*n* = 14, Table 1). Mele *et al*. (14) report that there was limited evidence of AEU between most human tissues. As discussed above, however, this may reflect specific features of the RNA-seq data and/or limitations of the methodologies available for identifying AEU. We revisited this issue by applying the **iGEMS** pipeline to pairwise comparisons to three of the human tissues in their analysis (adipose, blood and muscle).

Since cross tissue comparisons will include many examples where the entire transcriptional unit is expressed in only one tissue, we implemented an SD filter to remove genes based on a low and invariant expression signal. This SD step was used prior to step 1 of the iGEMS pipeline for analysis of the Affymetrix HTA 2.0 array across the paired comparisons. To select a suitable SD filter value (in this case SD = 3) we plotted the distribution of SD values. The relationship between expression and tissue identity was examined using PCA. We demonstrated that genes *preferentially* expressed in blood, separated blood samples from muscle and adipose (which co-clustered). GO analysis of the preferentially detected genes, for example blood versus muscle, confirmed that the gene lists contained the expected biological profiles (Supplementary Table S2). A biologically informative profile was apparent even when utilizing the lowest quartile for expression values for genes preferentially expressed in tissue suggesting that the SD threshold of three expression units for ‘detected’ left intact low expressed genes (Supplementary Figure S4). We detected a combined total of 25 338 genes with median expression above this background (>3 SD expression units) in human adipose, muscle and blood i.e. more than gene detection counts with RNA-seq in these three tissue, even when a favourable filter (towards RNA-seq) for ‘detection’ was considered (e.g. >0.1 RPKM in at least 25% of samples) see Supporting File 2: Supplementary Table S3.

**Figure 5.**
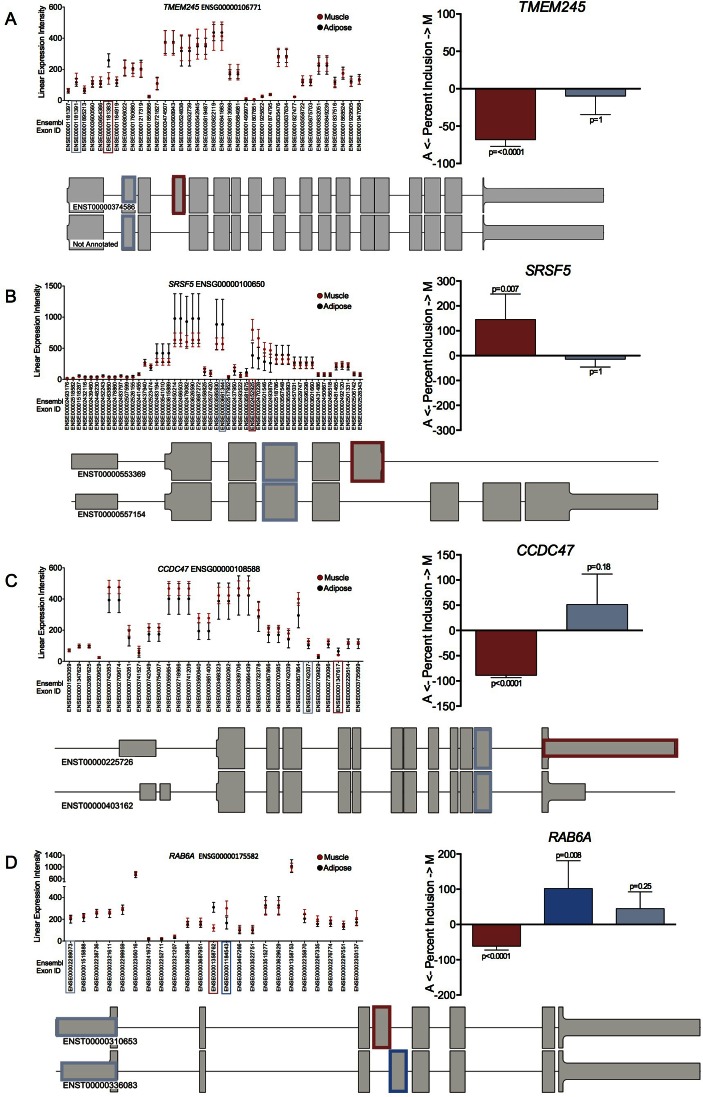
iGEMS identified diverse types of AEU events between muscle and adipose with RT-qPCR validation. We utilized the Affymetrix HTA 2.0 array to analyse tissue samples in a pairwise manner. Here we show four examples of different types of AEU events. Each accompanied with their respective linear expression intensities from the microarray analysis (mean ± SD). From a total of 14 pairs of twins, quality control validated paired microarray analysis was carried out on muscle (red, *n* = 12) and adipose (black, *n* = 12). RT-qPCR validation was carried out in remaining RNA (mean ± SD; muscle (*n* = 14) and adipose (*n* = 9)) and is shown as percent (%) change from adipose to muscle tissue with the colour consistent with the Exon ID plot on the left. (**A**) *TMEM245* (ENSG00000106771) undergoes an exon *exclusion* event in muscle as Ensembl Exon ID ENSE00001181383 is expressed much lower in muscle than adipose tissue. This AEU event was not evident in the ensembl database, but was represented in the NCBI database. (**B**) *SRSF5* (ENSG00000100650) undergoes an *alternative* 3′ UTR event in muscle and at least two *SRSF5* transcript variants exist: ENST00000557154 (full) and ENST00000553369 (truncated), with the latter transcript containing Ensemble exon ID ENSE00002452925 which was expressed in muscle to a greater extent. (**C**) *CCDC47* (ENSG00000108588) undergoes 3′UTR *extension* in adipose tissue since Ensembl exon ID ENSE00001347617 (probes span the entire 3′UTR) was detected in adipose more than muscle. (**D**) *RAB6A* (ENSG00000175582) undergoes a *mutual* splicing event which produces at least two transcripts, with each variant containing a mutually exclusive exon; ENSE00001358762 which is expressed more in adipose tissue (part of ENST00000310653) while the other exon (ENSE00001184543) was is expressed more in muscle tissue (part of ENST00000336083). Details on RT-qPCR validation applied to the human tissue can be found in Supplementary Figure legend S5 and Supporting Information 1.2.

In total there were 4421 genes with significant AEU events across pairwise comparisons of the three tissue types using the HTA 2.0 arrays (Supporting File 2: Supplementary Table S4). This is ∼5–10× more than Mele *et al*. observed in their pairwise analysis between the same tissues. To confirm that this reflected a high true positive rate we visually inspected AEU in 100 gene expression plots between muscle and adipose tissue with FDR values <5% and used RT-qPCR to validate AEU events (Figure 5 and Supplementary Figure S5, see Supporting File 2: Supplementary Table S5 for primers used). Most AEU events validated (∼95%) and involved of a variety of types of AEU events including exon inclusion/exclusion (Figure 5A), alternative 3′ UTR utilization (Figure 5B), 3′ UTR extension (Figure 5C) and mutual splicing events (Figure 5D). The very high validation rate in our study may reflect the conservative MUF and SI thresholds we selected. A proportion of genes had a significant MUF score but did not contain an exon with an extreme SI value (Supplementary Figure S6) but there were few true positives below our selected thresholds (according to visual inspection). Selecting an acceptable SI threshold will be study specific, in our case the criteria selected did not compromise identification of a high true positive rate and yields a high number of detectable events. We also noted that for muscle ∼15% of AEU events identified appeared to be driven by contamination of the muscle ‘signal’ by genes highly expressed in blood, rather than genes genuinely expressed in muscle (reflecting modest blood contamination of muscle biopsies).

**Figure 6.**
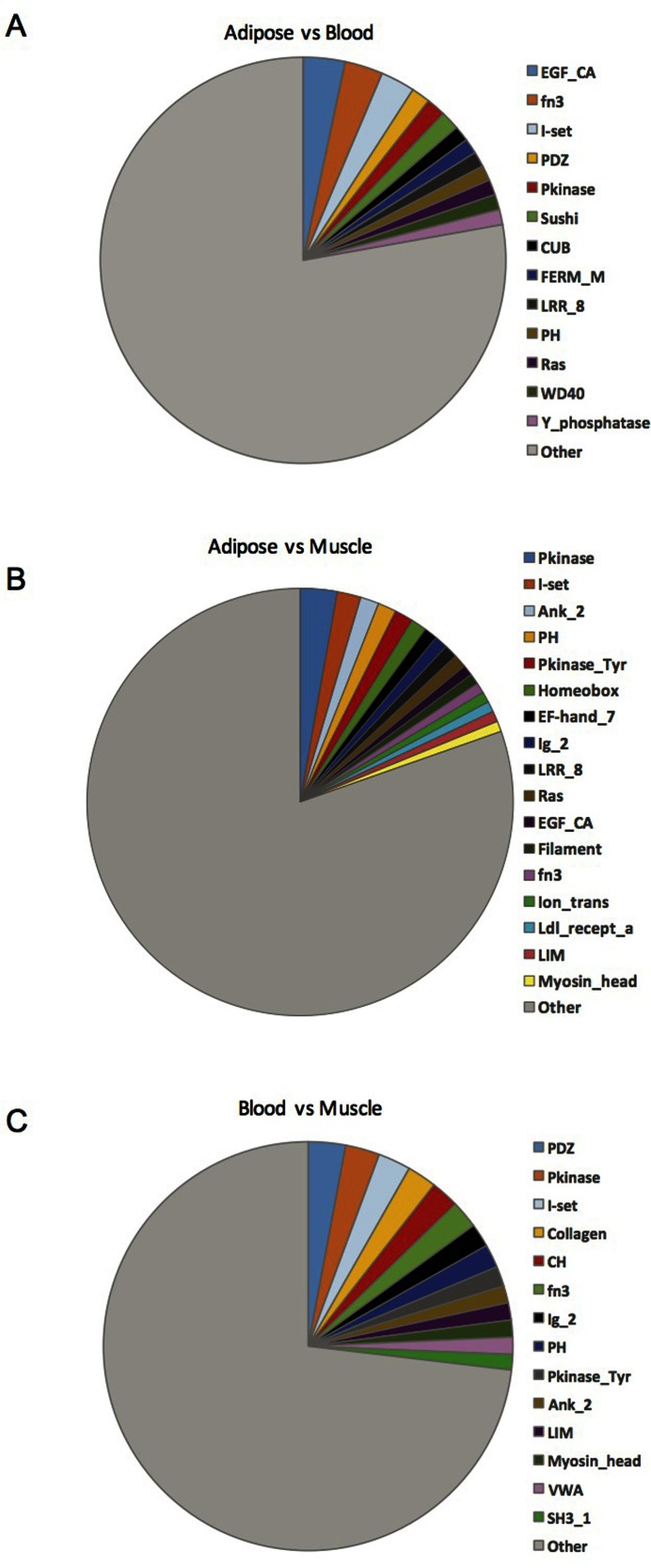
Frequency of Pfam domains altered by AEU across muscle, adipose and blood. Each chart represents a single pairwise comparison. Overlapping Pfam domains within Ensembl exon ID coordinates are counted. Only the most frequently occurring Pfam domain classes are shown individually while remaining classes are summed in the ‘other’ group. (**A**) For blood versus adipose, the Pfam domain is listed if it occurred ≥ 3. (**B**) For adipose versus blood, the Pfam domain is listed if it occurred ≥ 3. (**C**) For blood versus muscle comparison, the Pfam domain is listed if it occurred ≥ 9.

To characterize the potential impact of these events, genes undergoing AEU between tissues were subjected to GO analysis, identifying molecular functions associated with each pairwise comparison (Supporting File 2: Supplementary Table S6) e.g. actin binding and microfilament activity between muscle and blood. Further, protein domain analysis was performed to determine the frequency of Pfam domains present within exons undergoing AEU in each pairwise comparison (Figure 6), with the most common Pfam domains being individually listed (Supporting File 2: Supplementary Table S7).

## DISCUSSION

Alternate exon usage modifies protein biochemical function, diversifying canonical pathways and represents an important contributor to cell phenotype. Methods developed to detect differential splicing using DNA microarrays have lacked acceptable true positive rates, or clear guidelines for appropriate implementation. Here we introduce an AEU identification process that has a robust performance with minimal heuristics and robust control for multiple testing. Rather than limiting discovery of AEU events we demonstrate that exon focused DNA microarrays detect thousands of AEU events between human tissues, in contrast with analysis of the human tissue transcriptome using RNA-seq (14). As can be observed in Figure 6, a core of AEU events and modified Pfam categories are shared across each tissue pairing (e.g. muscle versus adipose and adipose versus blood) was substantial enough to suggest that AEU contributes substantially to tissue specialization. Within the limited number of genes with AEU identified by Mele *et al*. (14) <20% overlapped with the present analysis. Not unsurprisingly, the overlap was biased towards genes with greater than average RPKM units (Supplementary Figure S7), indicating that lack of robust quantification of RNA abundance by RNA-seq may explain the failure to note substantially amounts of AEU. In contrast, arrays identified AEU across the expression value continuum, with only a modest bias for genes with higher expression signal.

Using adipocytes treated with the anti-diabetic drug, rosiglitazone, *Agpat1* underwent AEU. *Agpat1* belongs to the 1-acylglycerol-3-phosphate O-acyltransferase protein family which are involved in phosphatidic acid production, which is an intermediate step in triglyceride synthesis (57). According to Ensembl, ENSMUSE00000934556 is a non-coding exon that appears exclusively in the shorter transcript: (ENSMUST00000173242, Figure 3C). Whereas ENSMUSE00000707276, the exon unique to the full variant, had a much lower CT (i.e. detected earlier in the RT-qPCR assay), consistent with the view that it is the dominant RNA variant (58). The regions absent in truncated *Agpat1* contain motifs III and IV, which are required for acyl-CoA binding and catalysis, and LPA binding (59). The shorter transcript (ENSMUST00000173242), with this alternative 5′ UTR, could yield a protein with distinct biochemical function due to loss of substrate binding site and hence loss of substrate level regulation of enzyme activity. Functional analysis of the inter-tissue analysis also revealed the expected biological narrative e.g. muscle included a variety of modifications to contractile proteins when compared with blood (Supporting File 2: Supplementary Table S6). Pfam domain analysis revealed that ‘EGF_C’ was most frequently modified functional motif between adipose and blood, while ‘PKinase’ protein kinase Pfam domain was common modified when comparing muscle with either blood or adipose tissue (Figure 6, Supporting File 2: Supplementary Table S7).

Previously published AEU pipelines appear optimized to single experimental designs or lack any laboratory validation. For example, PAC detects splice variants on the assumption that exon expression tends to follow the gene expression across the samples if there is no alternative splicing (22). PAC needs at least three distinct groups to derive a meaningful model and it fails when applied to the comparison of two conditions (28) and in our adipocyte experiment. Approaches such as the ‘SI method’ (20) and the conceptually similar MiDAS which uses ANOVA or ANOSVA which fits a linear model to the observed data with the aim of identifying non-zero interaction terms between sample groups and exons (23), have all been found to be unreliable. Critically, as the exon number increases the detection ability of statistical tests like MiDAS and ANOSVA decreases as they do not accurately correct for multiple testing (25).

IGEMS ranks genes both at a gene-level and at the exon level (Figure 1) providing direct information on which particular exon(s) are likely to be undergoing AEU within a gene that is first ranked to contain a significant AEU event by Step 1. Given that the HTA 2.0 array detected the expression of >25 000 ‘genes’ (coding and non-coding) across the three tissues profiled in the present study, it is clear that an automated pipeline is essential to study AEU using such technologies. IGEMS incorporates the FIRMAGene model (albeit with a modified statistic) using it for the first time with exon and ‘tiling’ type arrays. McGlincy *et al*. (60) used the original FIRMAGene method to examine the impact of the circadian cycle on AEU coupled with ANOVA to identify AEU. To improve true positive detection they used only the core probe-set (‘best’ annotated) map of the EXON microarray. However, they could only validate ∼50% of the highest ranked genes, by RT-qPCR. In contrast we validated >90% of our findings from the mouse EXON array using RT-qPCR (genes selected from the first 100 in order). Further extensive RT-qPCR of an additional 22 examples of AEU (with genes being selected from MUF scores ranked 18–1514th) from the HTA 2.0 twin tissue analysis yielded a 95% validation rate. This indicates that we have achieved a significant improvement in performance over all existing AEU analysis pipelines to yield a high true positive rate.

However, it is also important to consider why we were able to identify far more AEU between tissues than Mele *et al*. (14) using a similar number of biological samples. They determined when internal cassette exons were preferentially used with the ‘Percent Spliced-in’ (PSI) approach (61,62) applied to RNA-seq data. In doing so they concluded that variation in expression rather than variation in splicing explained tissue diversity. Despite their RNA-seq and our HTA analysis detecting a similar number of transcripts (14) we found ∼5–10× more AEU across human tissue types (their threshold for detection was rather generous). PSI in its simplest form is a ratio metric of reads that include the cassette exon versus reads that include or exclude the respective exon. Differences at a particular threshold on this ratio metric scale are considered to detect spliced exons. In comparison, the first step in iGEMS does not compare individual exons, but looks for small continuous changes along a gene. Also the PSI approach required two constitutively expressed flanking exons to identify the potentially spliced exon implying their method was biased towards central cassette exons, unlike iGEMS which identified a variety of AEU events, including alternative 3′ UTR utilization (Figure 5B). Further, since we observed that genes subject to AEU were also frequently (60–80% of those identified) differentially expressed, an accurate estimation of the contribution of expression versus AEU to inter-tissue variance is not necessarily a meaningful analysis, in our opinion. Clearly membership of each category would reflect the statistical thresholds chosen for AEU or differential expression in the experiment.

Data modelling considerations aside, we demonstrated that using a non-competitive detection system i.e. a DNA gene-chip (i.e. each converted/labelled ‘RNA’ is detected *independently* from each other) yields a rich view of the tissue transcriptome. Thus, DNA microarray technologies remain a very useful and complementary technology (12,13) to RNA-seq technologies, with the advantage that it is more reproducible (technical replicates *R*^2^ > 0.95 compared to *R*^2^ < 0.9 for RNA-seq (10,12)) and still more cost-effective, particularly in terms of data processing and data storage. Indeed, according to the SEQC the coverage and performance achieved with the latest HTA 2.0 DNA microarrays requires ∼100 million aligned reads per sample for the equivalent information to be yielded by RNA-seq (12). Given that Mele *et al*. report ∼80 million aligned reads per sample and still identified ∼5–10× fewer AEU events than the present study, this value for ‘equivalence’ may be a substantial under-estimate. For example, the number of read-alignments required for blood and muscle may substantially exceed 100 million, as we noted limited co-linearity between sequencing depth and number of detected low expression genes in blood and muscle tissue (Supplementary Figure S8). In conclusion, iGEMS is a robust methodology for detection of AEU events using global transcriptome data, yielding results with limited requirement for visual inspection and a high true positive rate. Our meta-analysis indicates that RNA-seq protocols do not provide sufficient quantification of much of the transcriptome (‘read coverage’), which limits alternative splicing analysis of RNA-seq data to only the highest abundance transcripts.

## Supplementary Material

Supplementary Data

SUPPLEMENTARY DATA
