# Supplementary material for: iGEMS: an integrated model for identification of alternative exon usage events
Source: Nucleic Acids Res. 2016 Apr 19;44(11):e109. doi: 10.1093/nar/gkw263 (PMC4914109; doi:10.1093/nar/gkw263)
Supplement: Supplementary Data [file gkw263_Supplementary_Data.zip › Supporting_File_1.pdf]

Supporting Figures

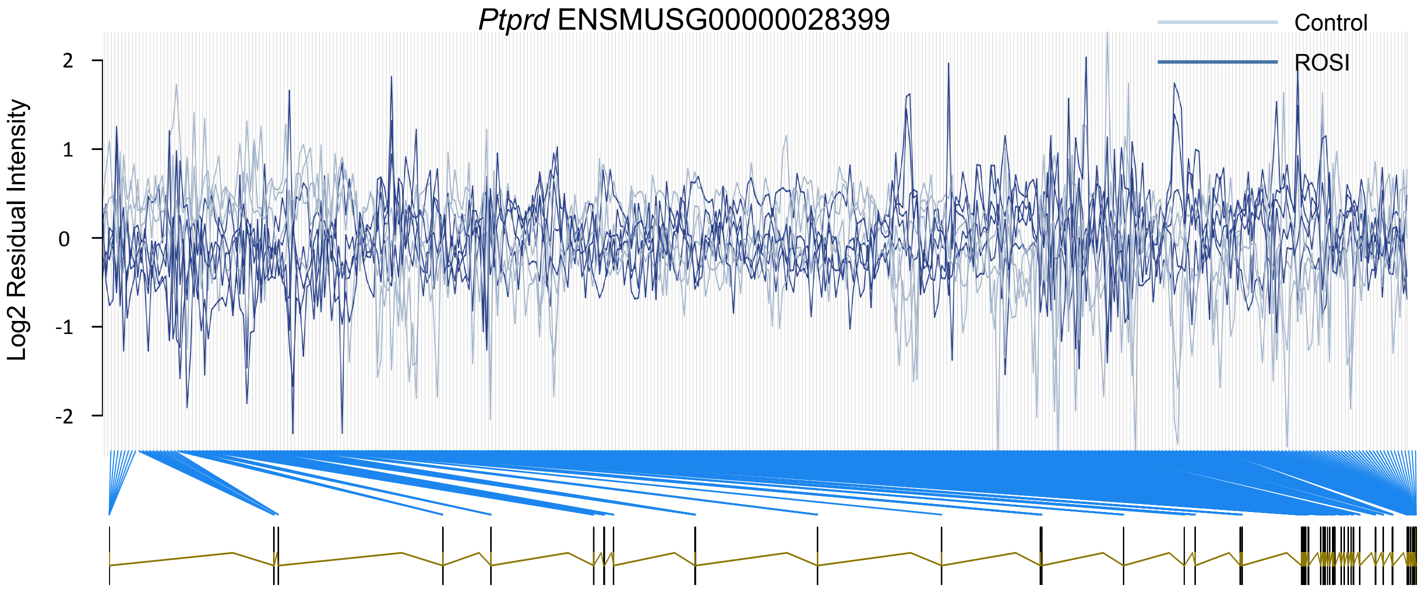

Figure S1: Residual plot of *Ptprd*. The figure shows that visual inspection of potential alternative exon usage (AEU) candidates is not always feasible in genes that have high number of exons. This emphasizes the need of having Step 2 within our pipeline to detect which exon within our candidate genes is undergoing an AEU event.

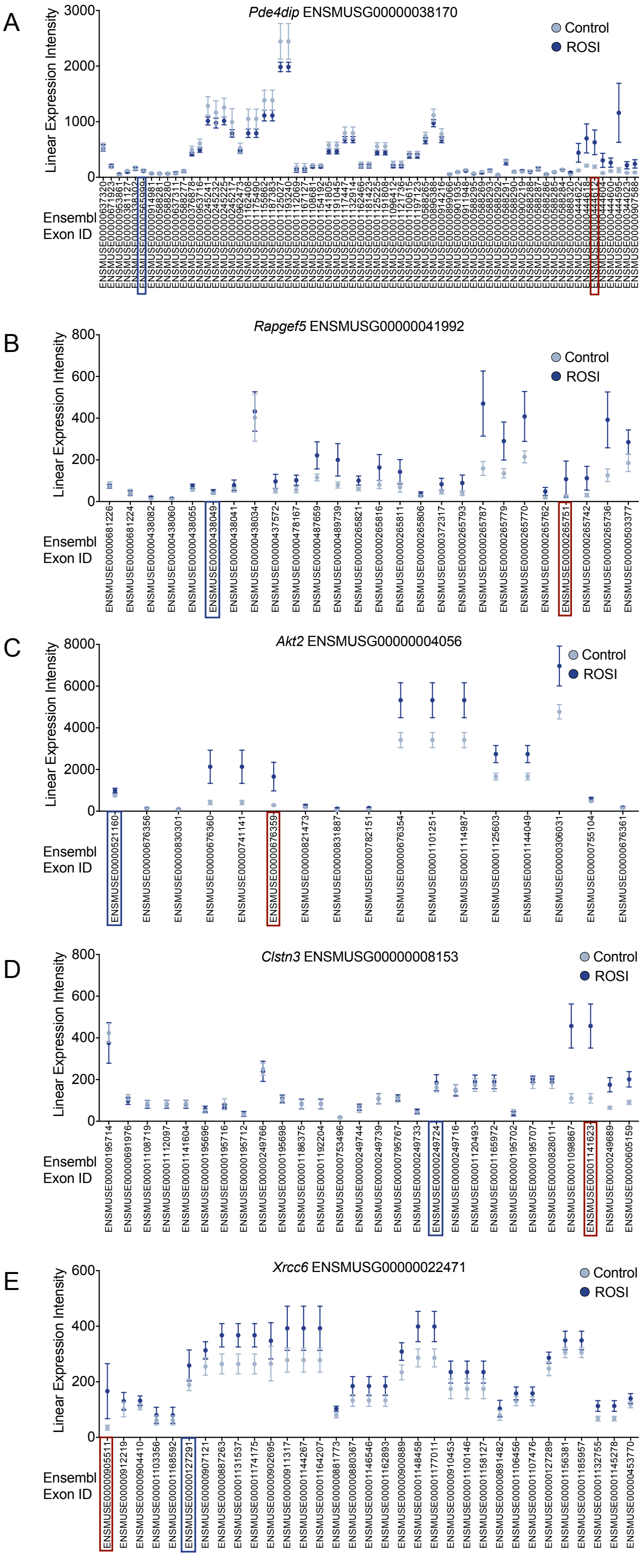

Figure S2: Expression intensities of genes undergoing an alternative exon usage (AEU) event in response to rosiglitazone (ROSI). A) *Pde4dip* (ENSMUSG00000038170), B) *Rapgef5* (ENSMUSG00000041992), C) *Akt2* (ENSMUSG00000004056), D) *Clstn3* (ENSMUSG00000008153) and E) *Xrcc6* (ENSMUSG00000022471). The blue and red box highlighting an Ensemble Exon ID denotes the constitutively expressed and AEU exon, respectively. Data is shown as mean ± SD (control group has n=8 and ROSI group has n=9).

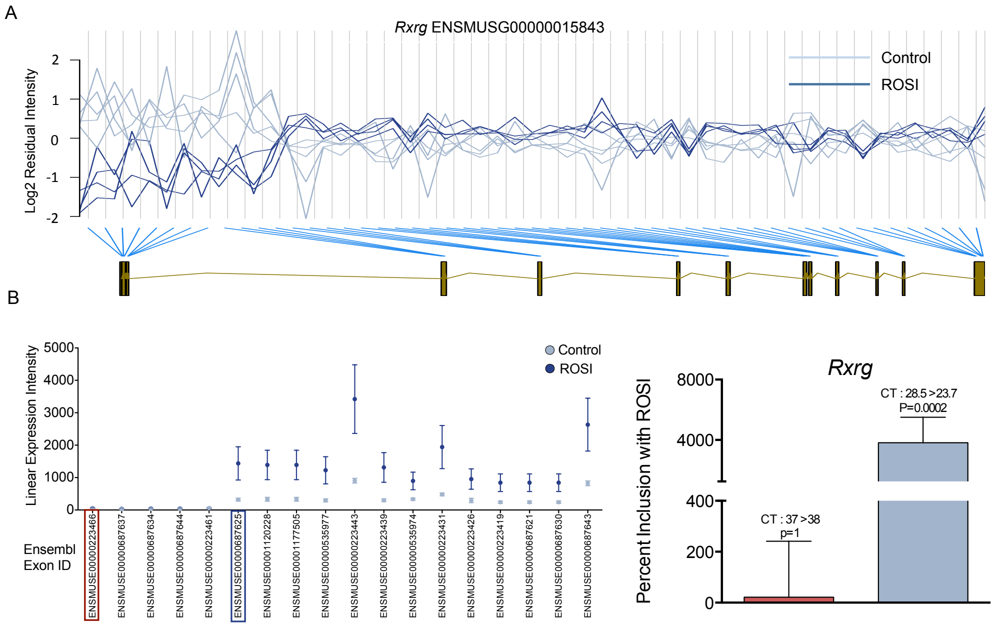

Figure S3: Identification of *Rxrg* as a false positive and is successfully removed by step 3 of iGEMS. A) A Material Unaccounted For (MUF) score is derived from the residual plot of *Rxrg* (ENSMUSG00000015843). The residual values were plotted in genomic order with the composite gene structure juxtaposed below. Our analysis was performed on 8 control (light blue) and 9 rosiglitazone (ROSI) treated cell cultures (dark blue). Blue lines connect the residual value to their respective genomic regions. On visualization we observed a large deviation from the model towards the 5' end of *Rxrg* indicating an alternative exon usage (AEU) event but this was a false signal that originated from exons that were near background expression. B) Expression plot of linear intensity (mean ± standard deviation (SD)) of each Ensembl Exon ID assigned to *Rxrg*; light blue represents the control and dark blue the ROSI treated values. Ensembl Exon ID: ENSMUSE00000223466 (red box) is the proposed AEU exon, whereas ENMSUSE00000687625 (blue box) is the apparent constitutively expressed exon according to splicing index. Primers were designed using this information. RT-qPCR validation was carried out in independent RNA (mean ± SD; control (n=8) and ROSI (n=8)) and is shown as percent (%) change from the control group with ROSI (mean ± SD) with the color consistent with the Exon ID plot on the left. CT values and adjusted p-values are shown for the control and ROSI group. RT-qPCR derived expression of the AEU exon did not change, whereas the constitutively exon increased significantly. Despite agreeing with the microarray data is not a genuine AEU event but rather represents gene expression showing step 3 is required in our pipeline.

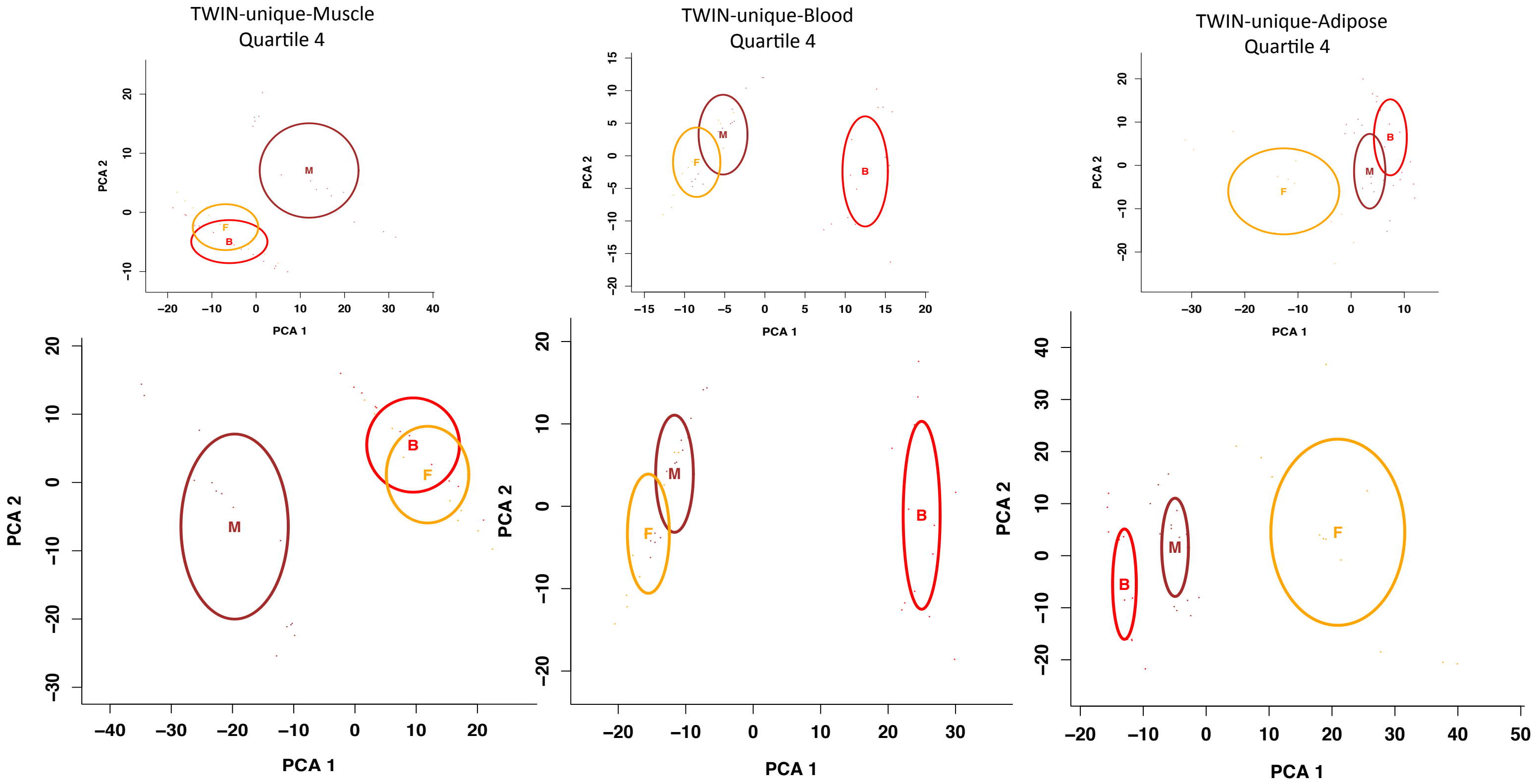

Figure S4: PCA ellipsoid plot for preferentially expressed genes in blood, muscle and adipose tissues respectively. The figure shows the genes expressed in a particular tissue, separates well from the remaining two tissues (which co-clustered). This biologically informative clustering was apparent even when utilizing the lowest quartile (quartile 4) of the tissue's preferentially expressed genes.

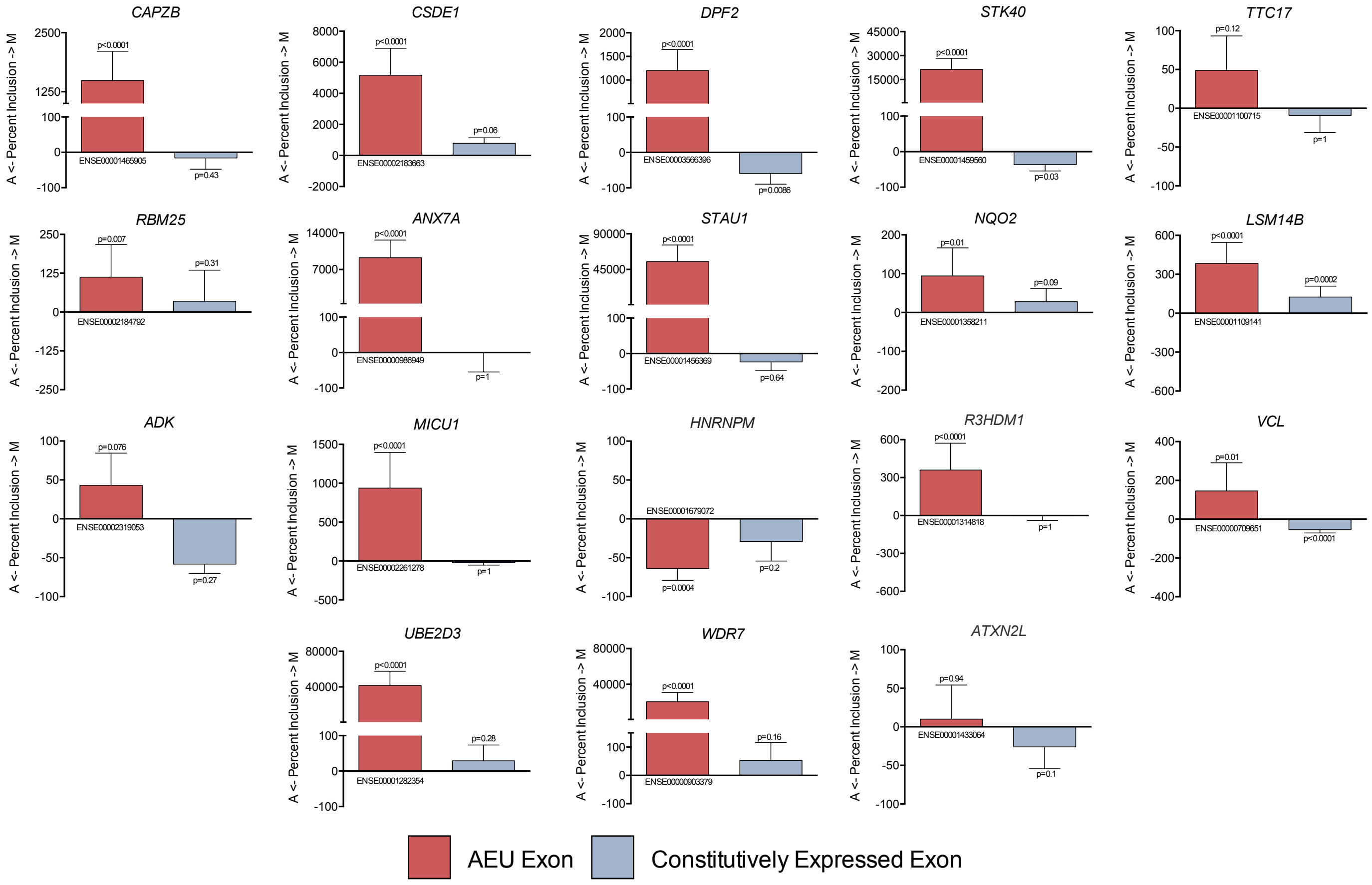

Figure S5: Summary of RT-qPCR validation of alternative exon usage (AEU) events between muscle and adipose tissue. IGEMS identified over 1500 genes with transcript variation between muscle and adipose tissue. Here we show validation of 18 genes undergoing an AEU event, all of which were successfully validate except for *ATX2NL*. It should be note that *UBE2D3*, *WDR7* and *ATX2NL* have FDR values within 5-8%. The RT-qPCR data is presented as percentage changes, where positive and negative shifts represent muscle (M) and adipose (A) inclusion, respectively. For each gene an AEU exon was measured and constitutively expressed exon for comparative purposes. Data is presented as mean ± standard deviation, muscle group has n=14 and adipose group has n=9. Above each bar plot the respective adjusted p-values are shown and above the AEU bar plot is the respective Ensembl Exon ID.

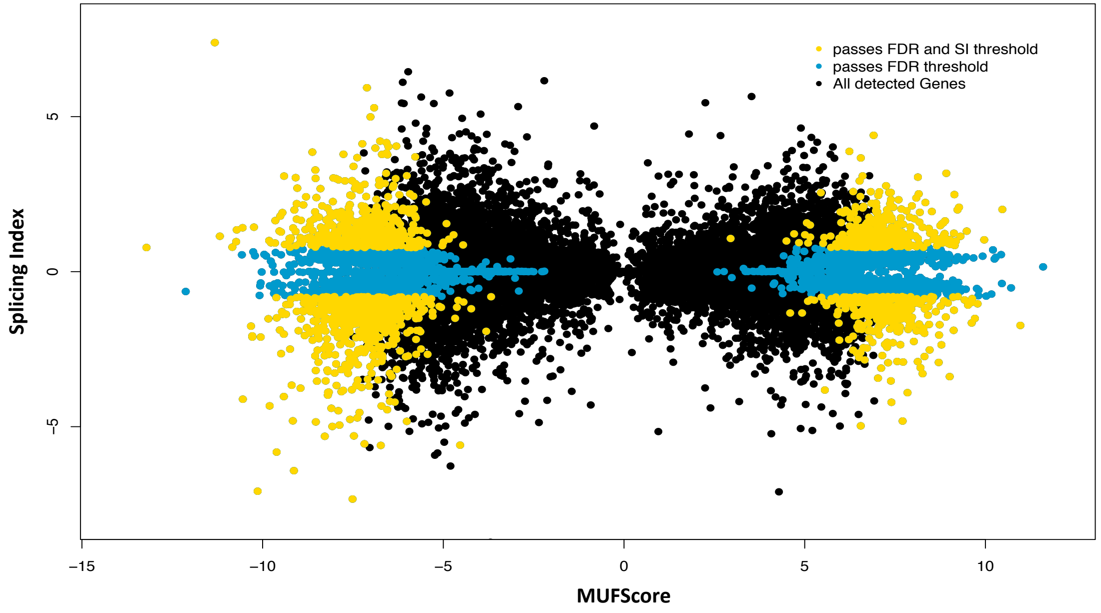

Figure S6: Relationship between Material Unaccounted For (MUF) score and splicing index (SI) in the comparison between muscle and adipose. Examination of the relationship between the MUF scores and SI identified few cases where modest MUF score at the gene level had evidence for a significant SI at the exon level and vice-versa. Here blue dots show genes that pass the FDR threshold (based on MUF score and gene size) but fails the 10% SI threshold and yellow dots show the candidates that pass both the FDR and SI threshold. The focus of our analysis strategy was to yield a high true positive rate without severely limiting the number of detectable events. The trade-off between true positives rate and SI boundary will ultimately be based on study specific characteristics.

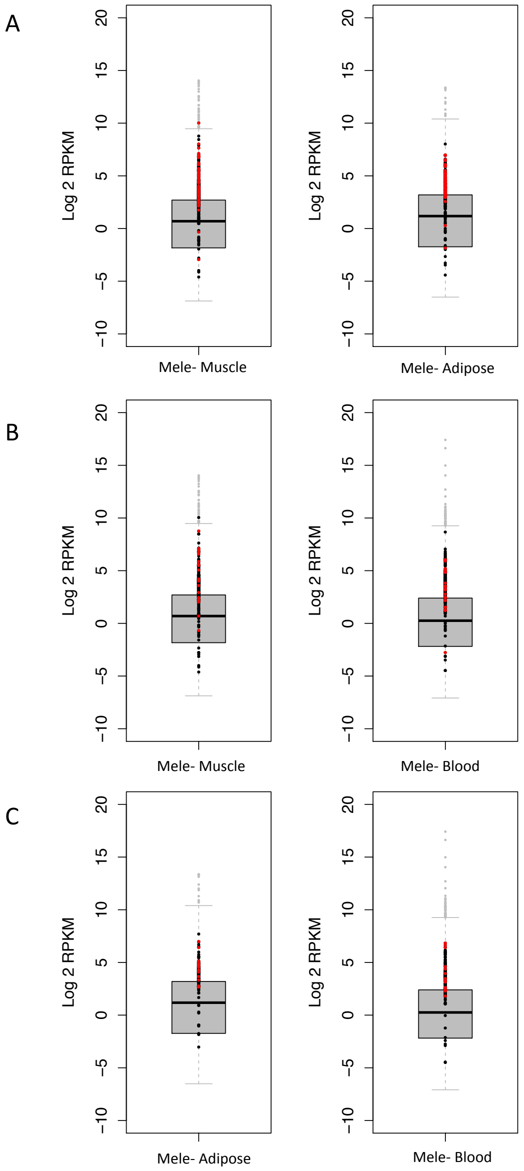

Figure S7: RNA-sequencing (RNA-seq) derived RPKM values from Mele *et al* [1] and their relationship to alternative exon usage (AEU). Data is shown for 3 pair-wise comparisons: A) Muscle vs Adipose B) Blood vs Muscle C) Adipose vs Blood. For each comparison two box plots are shown for both the tissues compared. Genes from Mele *et al* undergoing an AEU or not are shown as black dots and grey dots, respectively. Lack of robust quantification of RNA abundance by RNA-seq may explain failure in identification of significant AEU events. Majority of AEU events from Mele *et al* was observed in genes with higher RPKM values. Furthermore, the overlap with iGEMS (shown as red dots) was driven by genes with the highest median RPKM values.

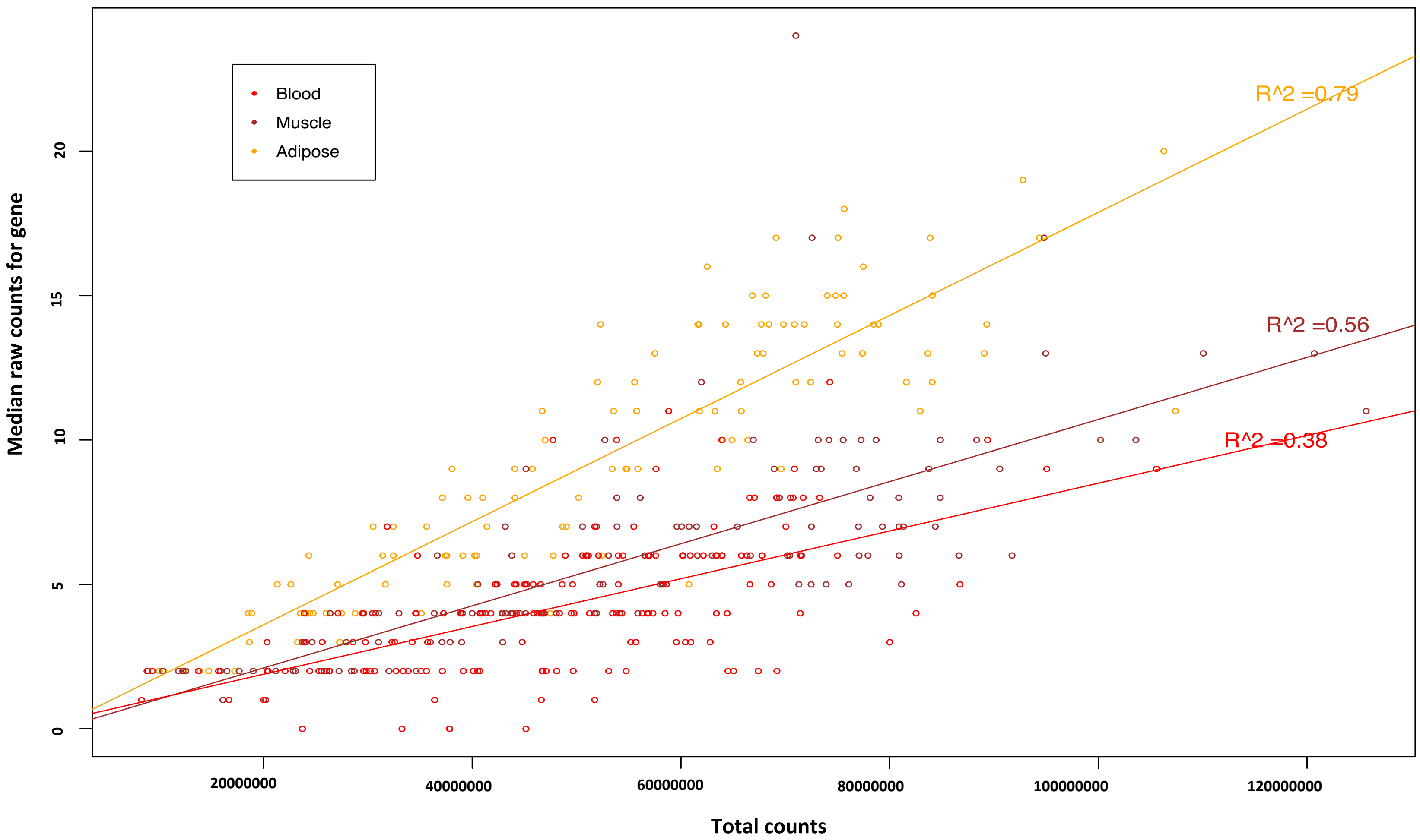

Figure S8: Relation between samples size and genes expressed at a low value as detected using RNA-sequencing (RNA-seq) technology. There was a very limited linear relationship between sequencing depth of low expressed genes in blood and muscle tissues while there was a more robust linear relationship in adipose tissue. This means that complete coverage of the transcriptome in muscle and blood would be very challenging and thus RNA-seq would not necessarily be preferential technology for detecting alternative exon usage events.

Supporting Information for iGEMS

1.1 Description of equations used in **i**ntegrated **G**ene **E**xon **M**odel of **S**plicing

A) Briefly, FIRMAGene [2] is built on the FIRMA [3] (Finding isoforms using robust multichip analysis) method that structures the problem of detecting alternative splicing as a problem of outlier detection. It believes that residuals from the RMA (robust multichip analysis) model holds the key to finding differential splicing events which is modeled according to the following equation:

$$y_{ijk} = c_j + p_k + error \qquad (1)$$

Where, $yijk$ represents log intensity of probe k of exon i measured in experiment j (signal has been background-corrected and normalized), cj is the experiment effect and pk is the probe effect.

The residual from the fitted RMA model describes the discrepancy of probe intensity in a given experiment from the expected expression and can be represented by the following equation:

$$r_{ijk} = y_{ijk} - \widehat{c_j} + \widehat{p_k} \qquad (2)$$

However unlike FIRMA, FIRMAGene does not focus on individual exons but instead interrogates exon regions (probes) that are adjacent in a gene to highlight persistence departure from the fitted model as an evidence of potential differential splicing.

When fitting a model RMA down-weights the probes that behave differently then the rest of the model in the fitting step. FIRMAGene takes these adjacent poorly fitting probes for a given sample as an evidence of differential splicing based on which it has devised a statistic called MUFScore (mean unaccounted for) to search for this behavior.

$$M_i^{(j)} = \max_{1<s<e<J} \frac{\left|\sum_{j=s}^{e} r_{ijk}\right|}{\sqrt{e-s+1}} \qquad (3)$$

Where, $M_i^{(j)}$is the maximum absolute partial sum over the J (J + 1)/2 possible consecutive sums of J probes within a gene and these denominator of this partial sum statistic has the square root of the number of data points. We performed this calculation for each gene on

the platform and associated a FDR value with each of the computed Material Accounted For score value. The FDR estimation is explained in the methods section of the paper.

B) Splicing index (SI) [4] is the ratio of exon level signal that has been normalized to the overall gene level signal. In the SI approach, exon inclusion rates under two conditions are compared to identify differential alternative splicing events. A significant difference in the normalized exon intensity indicates that the exon has different inclusion or exclusion rates (relative to the gene level) between the two conditions.

$$\text{Splicing Index (SI)} = \frac{(\text{Exon Intensity}_{i1}/\text{Gene Intensity}_{i1})}{(\text{Exon Intensity}_{i2}/\text{Gene Intensity}_{i2})} = log2\ (NI_{i1}\ /NI_{i2})$$

Where, NI_{i1} is normalized intensity for exon *i* in condition 1 and NI_{i2} is normalized intensity for exon *i* in condition 2. We used this method in our pipeline as an exon level detection filter to decide which exons within a candidate gene are undergoing a splicing event.

1.2 Experimental validation of Human AEU events by RT-qPCR.

For RT-qPCR 300 ng of total RNA was reversed-transcribed with the High-Capacity cDNA Reverse Transcription kit (Life Technologies) according to manufacturer's protocol using the same RNA on used on HTA 2.0, cDNA and was diluted 1:30 and 1 µl was added per well of the 384-optical well plates (Life Technologies). Exon specific primers (Sigma-Aldrich) were designed using Primer-BLAST for genes across a continuum of MUF scores. Primers were mixed with SYBR Select Master Mix (Life Technologies) and aliquots of 6 µl of mastermix were added to each well. Samples were run in triplicate. Thermal cycling conditions were 2 min at 50°C followed by 2 min 95°C and 40 cycles of 15 s at 95°C followed by 60 s at 60°C on a ViiA™ 7 Real-Time PCR System (Life Technologies, UK). Target mRNA expression was quantified using the ΔCT method [5]. To control for RNA input, 18s levels were measured using an Eukaryotic 18s rRNA Endogenous control kit (Life Technologies) according manufacturer's protocol using 1:4000 diluted cDNA. For each gene an AEU exon was contrasted to a constitutively expressed exon to determine if the AEU event is genuine. The data is presented as percent change (%) from adipose tissue. To determine the statistical significance a Mann-Whitney U test was preformed on the ΔCT values followed by

Bonferroni correction. Primers used for the HTA validation can be found in Supporting File 2: Table S5.

1.4 General RT-qPCR information.

Data was generated using ViiA7 RUO software (version 1.2.4) using default settings for CT choice, non-template control detection, multiple melt curve peak detection and outlier detection. GraphPad Prism (version 6.0) was used for statistical analysis. In this study 18s was used as the house keeping gene of choice for both studies as it has been shown to be consistent across two tissues [6]. No universal inhibition was found when a dilution curve was performed and samples were also checked for tissue identity using muscle (*DES*) and adipose (*FABP4*) gene marker genes. For each transcript specific RT-qPCR assay a serial dilution curve was performed at 5 descending concentrations (1:4, 1:16, 1:64, 1:256 and 1:1024) using fetal cDNA (Agilent Technologies and Takara Bio Europe Clontech), which were used to determine the limit of detection. Dilution curves were also used to calculate the gradient, PCR efficiency and R^2 for each assay (Supporting File 2: Table S1 and Table S5). In this study RNA integrity was not directly assessed but multiple primers used in this study measured the 5' or 3' end of a gene which produced adequate CT values, indicative of non-degraded intact RNA.

References

1. Melé M, Ferreira PG, Reverter F, DeLuca DS, Monlong J, Sammeth M, Young TR, Goldmann JM, Pervouchine DD, Sullivan TJ, Johnson R, Segrè A V, Djebali S, Niarchou A, Consortium TGte, Wright FA, Lappalainen T, Calvo M, Getz G, Dermitzakis ET, Ardlie KG, Guigó R: **The human transcriptome across tissues and individuals.** *Sci* 2015, **348** (6235):660–665.

2. Robinson MD, Speed TP: **Differential splicing using whole-transcript microarrays.** *BMC Bioinformatics* 2009, **10**:156.

3. Purdom E, Simpson KM, Robinson MD, Conboy JG, Lapuk A V, Speed TP: **FIRMA: a method for detection of alternative splicing from exon array data.** *Bioinformatics* 2008, **24**:1707–1714.

4. Srinivasan K, Shiue L, Hayes JD, Centers R, Fitzwater S, Loewen R, Edmondson LR, Bryant J, Smith M, Rommelfanger C, Welch V, Clark T a, Sugnet CW, Howe KJ, Mandel-Gutfreund Y, Ares M: **Detection and measurement of alternative splicing using splicing-sensitive microarrays.** *Methods* 2005, **37**:345–59.

5. Schmittgen TD, Livak KJ: **Analyzing real-time PCR data by the comparative CT method**. *Nat Protoc* 2008, **3**:1101–1108.

6. Bas a, Forsberg G, Hammarström S, Hammarström M-L: **Utility of the housekeeping genes 18S rRNA, beta-actin and glyceraldehyde-3-phosphate-dehydrogenase for normalization in real-time quantitative reverse transcriptase-polymerase chain reaction analysis of gene expression in human T lymphocytes.** *Scand J Immunol* 2004, **59**:566–573.
