# Supplementary material for: iGEMS: an integrated model for identification of alternative exon usage events
Source: Nucleic Acids Res. 2016 Apr 19;44(11):e109. doi: 10.1093/nar/gkw263 (PMC4914109; doi:10.1093/nar/gkw263)
Supplement: SUPPLEMENTARY DATA [file supp_44_11_e109__index.html]

iGEMS: an integrated model for identification of alternative exon usage events — SUPPLEMENTARY DATA 

# iGEMS: an integrated model for identification of alternative exon usage events

## SUPPLEMENTARY DATA

- SUPPLEMENTARY DATA
- SUPPLEMENTARY DATA
